# Better survival of older patients with stroke managed in a collaborative stroke pathway

**DOI:** 10.1007/s41999-025-01225-9

**Published:** 2025-06-27

**Authors:** Bruno Oquendo, Witold Jarzebowski, Charlotte Nouhaud, Anne Leger, Christel Oasi, Charlotte Havreng-Thery, Carmelo Lafuente-Lafuente, Joel Belmin

**Affiliations:** 1 Service de Gériatrie à Orientation Cardiovasculaire et Neuropsychogériatrique, APHP, Hôpitaux Universitaires Pitié-Salpêtrière-Charles Foix, Hôpital Charles Foix, 7 Avenue de la République, 94200 Ivry-sur-Seine, France; 2 Service de Gériatrie, Hôpital de Bastia, Bastia, France; 3 Service de Gériatrie, Hôpital Louis Mourier, Colombes, France; 4 Urgences Cérébro-Vasculaires, APHP, Hôpital Pitié-Salpêtrière, Paris, France; 5 Présage Care, Paris, France; 6 Sorbonne Université, Paris, France; 7 Laboratoire d’Informatique Médicale et d’Ingénierie des Connaissances pour la e-Santé (LIMICS), Paris, France; 8 Clinical Epidemiology and Ageing (CEpiA), Unit Institut National de la Santé et de la Recherche Médicale (INSERM), Créteil, France

**Keywords:** Stroke, Geriatrics, Older adults, Collaborative care pathway, Rehabilitation, Mortality

## Abstract

**Purpose:**

To compare the survival of stroke patients over 70 years old managed in the collaborative Stroke Pathway dedicated to the OLD patients (SPOLD) with those referred to conventional rehabilitation wards.

**Methods:**

This longitudinal observational retrospective cohort study involved over 70 years old patients referred from the same neurovascular emergency unit in a university hospital in France to a post-stroke geriatric unit within the SPOLD pathway, compared to patients referred to conventional rehabilitation ward during the same period. Initial stroke severity and comorbidities were assessed using the NIHSS score and the Charlson index, respectively. The primary endpoint was 2-year survival, analyzed using Cox models for both the entire cohort and a propensity score-matched cohort to control for referral bias.

**Results:**

The study included 262 patients with a mean age of 84.5 ± 6.7 years, of whom 122 (46.6%) were male. Patients in the SPOLD group (*n* = 101) had significantly higher age, NIHSS scores, and Charlson indices compared to those in the conventional rehabilitation ward (*n* = 161). Adjusted mortality was significantly lower in SPOLD patients (OR: 0.525, 95% CI: 0.298 to 0.924, *p* = 0.025). In the propensity score-matched cohort, mortality was also significantly lower for SPOLD patients (OR: 0.426, 95% CI: 0.212 to 0.857, *p* = 0.017).

**Conclusion:**

This study suggests that this collaborative organization between a neurovascular emergency unit and a rehabilitation geriatric unit may be associated with a better survival of older patients after stroke.

**Supplementary Information:**

The online version contains supplementary material available at 10.1007/s41999-025-01225-9.

## Key summary points

**Aim:**

To investigate short- and long-term post-stroke mortality in patients in a collaborative Stroke Pathway dedicated to the OLD patients (SPOLD).

**Findings:**

We observed longer 2-year survival in patients managed as part of a collaborative care pathway including neurologists and geriatricians, compared with those referred conventionally. This result was significant after reducing biases related to age, gender, stroke severity score, as well as disability, cognitive impairment and comorbidity levels, using cohort matched on propensity scores.

**Message:**

This study suggests that collaboration between neurologists and geriatricians with a dedicated pathway may improve stroke survival in older patients.

**Supplementary Information:**

The online version contains supplementary material available at 10.1007/s41999-025-01225-9.

## Background

Stroke is the second leading cause of death worldwide [1]. Between 1990 and 2019, the global number of ischemic stroke deaths increased from 2.04 million to 3.29 million and is expected to increase to 4.90 million by 2030 due to age and the global burden of the risk factors [2]. The incidence of stroke doubles in 10-year increments after the age of 55 for both men and women [3]. Worldwide, 69% of people with stroke were over 65 years of age [1]. Stroke-related mortality is severe, even if major advances have been achieved by emergency revascularization of acute stroke [4].

Managing stroke in the very old is a challenge [5]. These people have more limited access to revascularization, are frailer, have complex comorbidities and pre-existing disabilities [6–10]. As a result, short- and long-term mortality is higher after hospitalization for stroke in patients over 85 [11, 12]. In addition, a higher proportion of very old stroke survivors require human assistance with activities of daily living and/or are admitted to long-term care facilities [5, 13]. Rehabilitation of these patients after stroke is complex, and requires a skilled team capable of managing the consequences of stroke as well as geriatric syndromes and various chronic and acute comorbidities [14, 15]. In our setting, we developed a partnership between neurologists in a neurovascular emergency unit and a geriatric team, resulting in a structured hospital care pathway to facilitate referral of very old patients with acute stroke to a geriatric unit, named in English Stroke Pathway dedicated to the OLD patients (SPOLD). This pathway has already shown benefits for patients in terms of functional recovery at three months and a reduction in total length of stay [16]. To our knowledge, this model of care is innovative, as we found no reports of similar experiences in the literature.

The objective of the study was to investigate short- and long-term post-stroke mortality in patients over 70 years of age who had benefited from SPOLD.

## Methods

### Design and settings

This longitudinal observational retrospective cohort study was carried out on stroke patients (> 70 years) admitted in one neurovascular emergency unit of a university hospital in France. On discharge, patients were referred either to a collaborative pathway with a geriatric ward, the post-stroke geriatric unit, or to conventional rehabilitation wards. Referral to the post-stroke geriatric unit or conventional rehabilitation ward (CRW) was not randomized, but was made primarily on the basis of bed availability and the patient’s place of residence, in line with usual practice. All referral units were located within a restricted perimeter around the neurovascular unit. Second, the main criteria for referring patients to the post-stroke geriatric unit were the complexity of the patients, who would then normally be referred to a geriatric unit, as a usual practice. This complexity was linked to multiple comorbidities and the management of associated pathologies other than stroke.

The post-stroke geriatric unit comprised a geriatric acute care unit and a geriatric rehabilitation unit that provide care in accordance with the principles of geriatric medicine, including multidimensional geriatric assessment, multidisciplinary management of geriatric syndromes and early consideration of social issues. Prior to its opening, the medical staff of both the neurovascular unit and the geriatric unit met collaboratively to define priorities: detection and management of stroke complications, complete the etiological assessment of strokes, in particular the search for atrial fibrillation, detection and management of geriatric comorbidities, performing a classical geriatric assessment, reviewing medication, optimizing rehabilitation, social care, organizing discharge to home care or institutionalization, and training paramedical staff in post-stroke rehabilitation. We did not define a specific age threshold for admission to the post-stroke geriatric unit, but it was clear to the neurologists and geriatricians involved in the SPOLD that very old and complex patients were welcome in the post-stroke geriatric unit. Compared to a classic geriatric unit in France with the same number of beds (12 beds in the geriatric acute care unit and 12 beds in the geriatric rehabilitation unit), it was necessary to add a part-time physiotherapist to take care of the patients three times a week. So, one physiotherapist was dedicated to the geriatric part of the pathway. The presence of a psychomotor therapist and an occupational therapist at 40% capacity had to be ensured, as well as the intervention of a dietitian and a speech therapist in the unit. These professionals were shared among several services, but all received training in the management of post-stroke patients, as in the control group. Intensity and frequency of therapy is adapted to the patient need and fatigability. The rehabilitation resources are not superior to those used in the control group. A pharmacist was present in the hospital but is not dedicated to the service. The nurses and nursing assistants in the pathway were working in the same geriatric department before the implementation of the pathway and received training for post-stroke rehabilitation care. There were no architectural modifications or purchases of expensive equipment in the post-stroke geriatric unit to operate the pathway.

The CRW considered in this study corresponded to usual referral wards for stroke patients of this neurovascular emergency unit prior the creation of the SPOLD dedicated to the old patients. They comprised rehabilitation units for all adults, not just the older ones. These units are located either in public hospitals (90.1%) or private hospitals (9.9%). In CRW, patients were treated by a physiotherapist four to five times per week. Most of these centers had expertise from speech therapists, psychomotor therapists, dietitians, and occupational therapists as part of post-stroke rehabilitation. These four paramedical professionals were not available full-time in most of the conventional care wards. Care was primarily provided by rehabilitation physicians. There was no pharmacist dedicated exclusively to these services. The number of nurses and nursing assistants was similar to the post-stroke pathway. Care revolved around the diagnosis of impairments and disabilities, the development and supervision of personalized therapeutic programs, and the coordination of multidisciplinary teams. Rehabilitation of post-stroke deficits was at the core of care in this ward. Conventional rehabilitation ward benefited from greater allocation of physiotherapists and more comprehensive technical facilities. There was no geriatrician involved in the conventional ward in this study.

In the event of a complication, each unit is managed in line with best practice recommendations. Depending on the complications, patients were treated in the referral unit or transferred to another unit if an interventional procedure was required, or if intensive care or resuscitation was indicated. In both groups, patients were discharged from hospitalization after reaching a rehabilitation plateau, with no further functional progress observed. In France, the services in both groups are funded by the French social security system, and no treatment in either group can be stopped due to a lack of funding.

### Participants and measurements

All the patients consecutively hospitalized in the neurovascular unit for a stroke between January 1, 2013 and January 1, 2017 were eligible. Inclusion criteria were age over 70, admission for stroke (ischemic or hemorrhagic) and referral to a rehabilitation service after the acute phase. Non-inclusion criteria were subarachnoid hemorrhage, death during hospitalization and discharge home after the acute phase.

For each participant, we recorded from the hospital unit electronic files all the following measurements: age, sex, medical history (prior stroke, hypertension, diabetes, myocardial infarction, atrial fibrillation), and social conditions before hospital admission (living at home or not, requiring assistance or not for activities of daily living, presence or not of a relative).

We also recorded information about stroke severity, comorbidities and functional status at baseline. On admission to the neurovascular unit, stroke severity score was systematically assessed by the NIHSS scale (supplementary material 1). The initial NIHSS is predictive clinical progression at 3 months [17, 18]. We also calculated for each participant the Charlson index from all the diseases listed in the medical record. This index has been widely used in geriatric studies and is predictive of survival [19, 20]. We also obtained information about functional independence from the Rankin score systematically assessed in the neurovascular unit within 48 h of entering in the neurovascular. The Rankin score (supplementary material 2) assesses overall functional independence from pre-stroke activities. Cognitive impairment was assessed by the Mini-mental status examination (MMSE) carried out during the patient’s hospital stay at a distance from the stroke within 8 days of entering the discharge unit. A MMSE score of less than 24 defined cognitive impairment [21].

The outcome, the survival status, was determined by several means. First, we reviewed the follow-up recorded in the electronic medical records, and then called the patients or their proxies. For missing information, we wrote to the civil registries of the town halls to find out the vital status and date of death of the deceased. The primary endpoint was the difference in survival within 24 months after stroke between the SPOLD and CRW groups.

### Statistical analysis

We compared the two groups for category variables using the Chi2 test and quantitative variables by the Student’s t test. Unadjusted and adjusted mortality hazard ratios of the SPOLD patients were calculated by Cox proportional hazards models using CRW patients as the reference. Adjustment variables were age, NIHSS severity score, initial Rankin score, MMSE score and Charlson index. Survival curves were plotted using a Kaplan–Meier method and compared by the logrank test.

To control for bias related to type of referral (SPOLD or CRW), we have also studied odds ratios for mortality in a propensity score–matched cohort. Cases patients were matched with controls on their propensity score by nearest neighbor method using two neighbors for one case, within a caliper of 0.005 SD. Variables used for matching allocation age and NIHSS severity score. A standardized mean difference (SMD) < 0.20 for all matching variables was considered as satisfactory balance. Unadjusted mortality odds ratios were calculated by Cox proportional hazards models and survival curves were plotted using a Kaplan–Meier methods and compared by the logrank test.

The statistics were produced using Stata software v16.1 (StataCorps, USA). The level of significance was a *p* value < 0.05.

### Ethics

The study was conducted according to the principles of the Declaration of Helsinki and the French current law. The protocol has been submitted and approved by the local ethical research committee (CER-22-067). The study was waived from participant consent due to its retrospective nature, in compliance with French regulations for this type of study. In those hospitals, all patients were given at admission written information about the possible use of their medical data for research purposes and agreed that they may be contacted at a later date to know their evolution and that they have the right to refuse at any moment to this use of their data. Data were de-identified before being used in the statistical analysis file.

## Results

### Participants

**Fig. 1:**
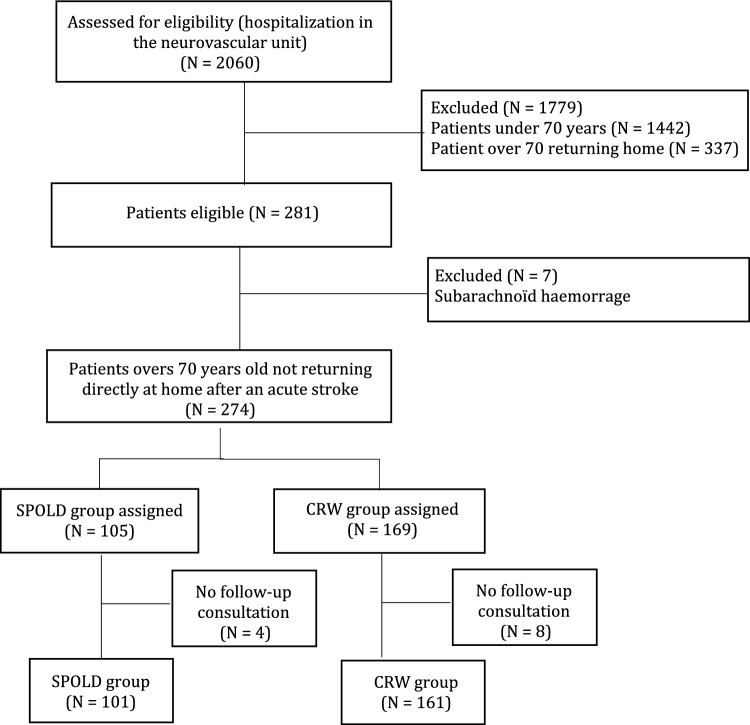
Flowchart of the study. SPOLD, stroke pathway dedicated to the OLD patients; CRWs, conventional rehabilitation wards

Of the 281 eligible patients, 262 patients were included in the study comprising 101 patients referred in the SPOLD and 161 in the CRW (Fig. 1). For several variables, values recorded at admission differed significantly between the two groups. As compared to CRW patients, SPOLD patients were older (*p* < 0.001) and had a greater comorbidity score (*p* < 0.001); hypertension, myocardial infarction and atrial fibrillation were significantly more frequent (*p* < 0.001 for the three diseases). They were significantly more dependent with a higher presence of assistance at home before hospital admission (*p* < 0.001). Their NIHSS score was significantly greater (*p* < 0.001), that indicates a higher stroke severity. Patients in the SPOLD were functionally more disabled with a higher initial Rankin scale (*p* < 0.001). (Table 1).

**Table 1:** Characteristics of the patients recorded at admission in the neurovascular unit in patients managed in the stroke pathway dedicated to the OLD patients (SPOLD) or to those referred to the conventional rehabilitation wards (CRWs)

Population	SPOLD group (*n* = 101)	CRW group (*n* = 161)	*p*
Age in years, m (SD)	84.5 (6.0)	79.6 (6.4)	< 0.001
Males, n (%)	41 (40.6)	81 (50.0)	0.161
Charlson’s index, m (SD)	7.48 (2.04)	6.50 (1.92)	< 0.001
Medical history, n (%)			
Prior stroke	24 (23.8)	33 (20.5)	0.643
Hypertension	80 (79.9)	84 (52.1)	< 0.001
Diabetes	28 (27.8)	33 (20.5)	0.232
Myocardial infarction	18 (17.8)	12 (7.4)	0.004
Atrial fibrillation	44 (43.6)	36 (22.4)	< 0.001
Living at home, n (%)	98 (97)	160 (99.4)	0.324
Helping (> 10 h), n (%)	35 (34.7)	20 (12.4)	< 0.001
Presence of a relative, n (%)	55 (54.6)	58 (36.0)	0.051
Ischemic stroke, n (%)	86 (85.1)	134 (82.7)	0.816
NIHSS score, median (IQR)	10 (4 to 18)	6 (3 to 12)	0.004
Rankin score, m (SD)	3.73 (0.90)	3.26 (0.79)	< 0.001
Cognitive impairment^a^, n (%)	36 (59.0)	48 (39.7)	0.012
Length of stay	67.09 (55.5)	51.78 (52.5)	0.120

^a^98 missing values

### Survival analysis

**Fig. 2:**
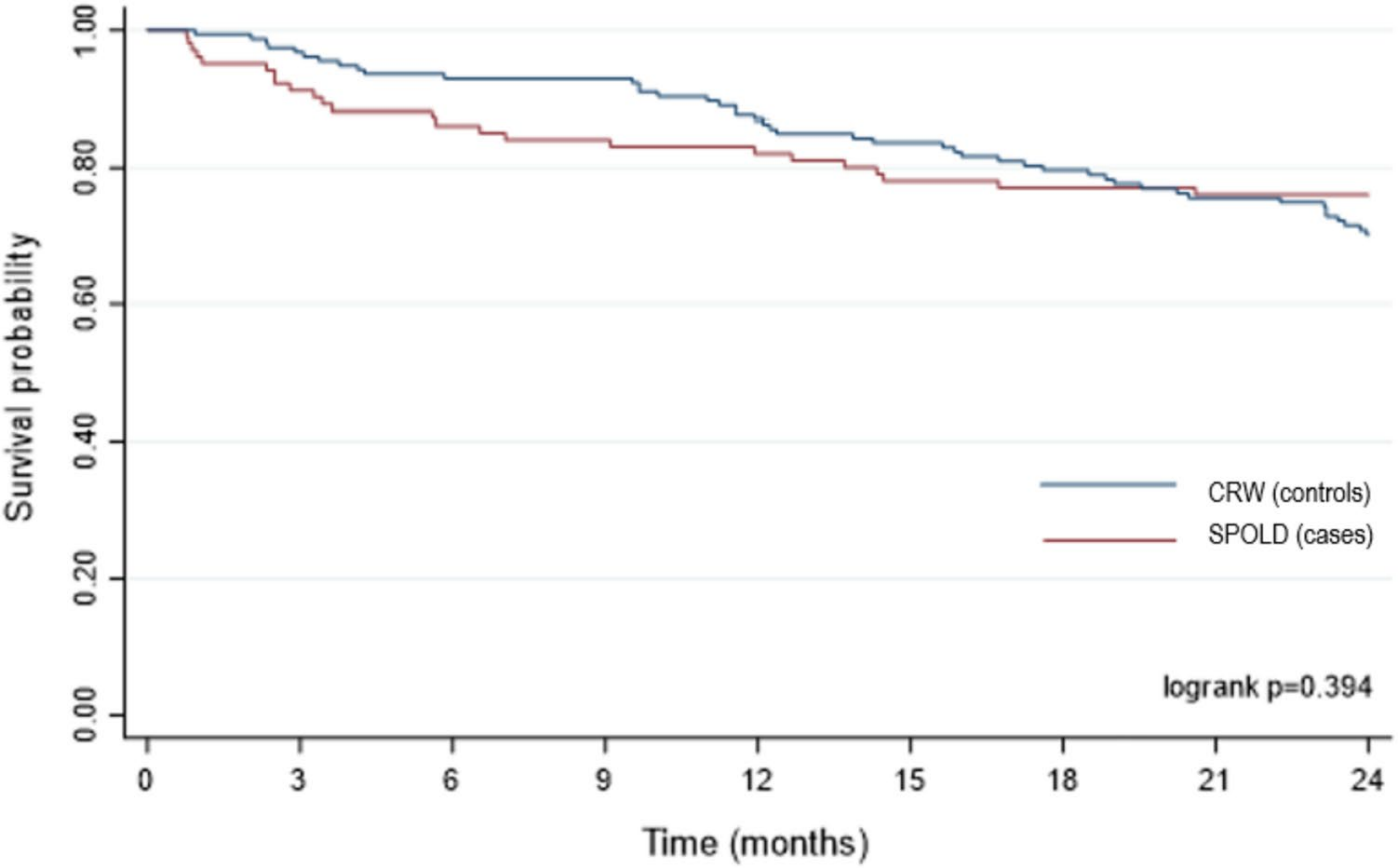
Survival plot for post-stroke patients over 70 years of age within 2 years of stroke. SPOLD, stroke pathway dedicated to the OLD patients; CRWs, conventional rehabilitation wards

The survival curves for the 2 years following stroke are shown in Fig. 2. There was no significant difference between the curves of the two groups (*p* = 0.394).

Unadjusted odd ratio for mortality was not significantly reduced in the SPOLD patients as compared to CRW patients, (OR: 0.81, 95% CI: 0.495 to 1.320, *p* = 0.395). The adjusted odds ratio for mortality was significantly reduced in SPOLD patients (OR: 0.525, 95% CI: 0.298 to 0.924, *p* = 0.025) (Table 2).

**Table 2:** Odd ratios for mortality at 24 months after a stroke on patients over 70 adjusted on age, gender, NIHSS severity score, initial Rankin score, presence of cognitive impairment and Charlson’s co-morbidity index

Factors studied	Odd ratio	95% CI	*p*
CRW (reference)			
SPOLD group	0.525	(0.298 to 0.924)	0.025
Age	1.026	(0.986 to 1.069)	0.205
NIHSS	1.034	(1.003 to 1.067)	0.032
Charlson index	1.081	(0.958 to 1.219)	0.204
Initial Rankin score	1.177	(0.872 to 1.589)	0.286
Cognitive impairment	0.691	(0.373 to 1.251)	0.222

CRWs, conventional rehabilitation wards; SPOLD, stroke pathway dedicated to the OLD patients; NIHSS, National Institutes of Health Stroke Scale

**Fig. 3:**
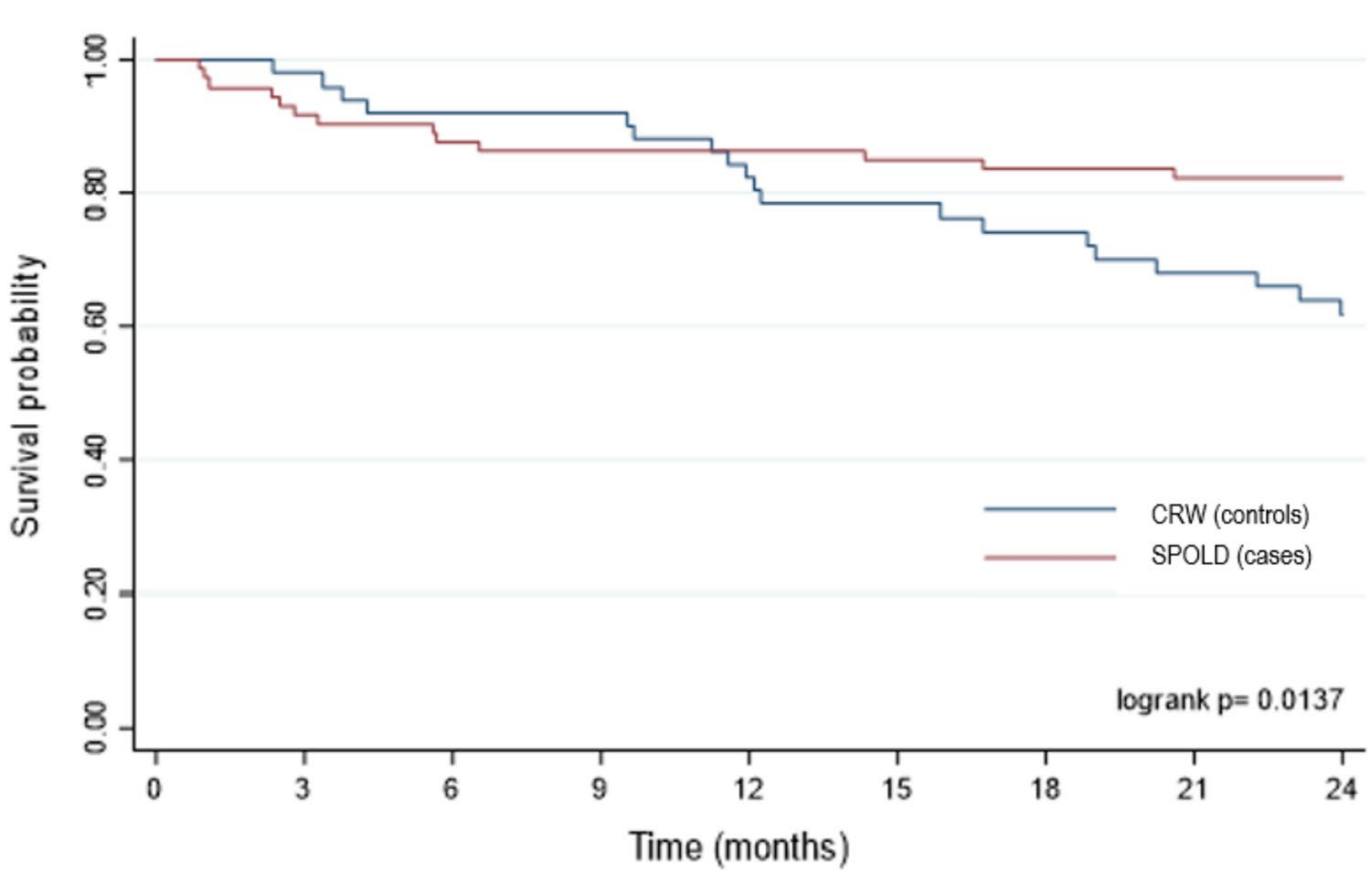
Survival plot for post-stroke patients over 70 years of age within 2 years of stroke on the propensity-matched cohort. SPOLD, stroke pathway dedicated to the OLD patients; CRW, conventional rehabilitation

To reduce the influence of bias due to differences in patient’s characteristics or referral, we performed a propensity-matched cohort analysis. In the propensity match cohort of 127 patients (74 in the SPOLD group and 53 in the CRW group). For matching we used the two variables with the largest differences between groups in the whole cohort, namely age and NIHSS score. In the propensity score-matched cohort, the SMD for these variables were small and < 0.20: 0.017 and 0.029 for age and NIHSS score respectively, indicating a satisfactory balance. The characteristics of the two groups retained in the propensity score-matched cohort are shown in the Table 3. Survival plots were significantly different with a better survival in SPOLD patients (Fig. 3, *p* = 0.01), and the unadjusted odd ratio for mortality was significantly reduced (OR: 0.433, IC95%: 0.191 to 0.982, *p* = 0.045) (Table 4).

**Table 3:** Characteristics of the patients in the propensity-matched cohort

Patients with stroke (*n* = 127)	SPOLD group (*n* = 53)	CRW group (*n* = 74)	Standardized mean difference
Age (in years)	82	81	− 0.15
Sex (male, %)	52.2	47.8	0.15
NIHSS (m)	9.28	9.09	0.20
Charlson index (m)	5.41	6.30	− 0.18
Rankin after stroke (m)	3.43	3.47	− 0.05
Cognitive impairment (%)	30.4	45.6	− 0.08

SPOLD, stroke pathway dedicated to the OLD patients; CRW, conventional rehabilitation wards; NIHSS, National Institutes of Health Stroke Scale; m = mean

**Table 4:** Odd ratios for mortality at 24 months after a stroke on patients over 70 adjusted on age, gender, NIHSS severity score, initial Rankin score, presence of cognitive impairment and Charlson’s co-morbidity index

Factors studied	Odd ratio	95% CI	*p*
Conventional rehabilitation wards (reference)			
SPOLD group	0.433	(0.191 to 0.982)	0.045
Age	1.026	(0.983 to 1.081)	0.205
NIHSS	1.034	(0.974 to 1.056)	0.032
Charlson index	1.081	(0.679 to 2.176)	0.204
Initial Rankin score	1.177	(0.796 to 1.652)	0.286
Cognitive impairment	0.691	(0.650 to 1.340)	0.222

SPOLD, stroke pathway dedicated to the OLD patients; NIHSS, National Institutes of Health Stroke Scale

## Discussion

In this longitudinal study of older patients with stroke, we observed longer 2-year survival in those managed as part of a collaborative care pathway including neurologists and geriatricians, compared with those referred conventionally. This result was significant after reducing biases related to age, gender, stroke severity score, as well as disability, cognitive impairment and comorbidity levels, using cohort matched on propensity scores.

To our knowledge, this study is the first to evaluate the contribution of geriatric management to the long-term survival of patients aged over 70 who have had a stroke. Management of patients with cerebrovascular pathology in the medium to long term is well developed but not in geriatrics field with a pathway dedicated to the older adults [22–26]. The post-stroke geriatric unit itself is an original concept and we did not find in the scientific literature any report of similar geriatric unit. The better survival observed in our study is in consistent with short-term results observed in the same cohort. Functional recovery of all these patients was recorded at 3 months and we observed that the Rankin scale values were significantly less in the patients managed in the SPOLD as compared to those in conventional care [16]. In addition, we observed a significant reduction in total length of hospital stay in the patients managed in the SPOLD [16]. Taken together, the results of our two studies converge and support the idea that this collaborative care pathway improves the prognosis of older patients with stroke.

The value of this care pathway lies in geriatric care optimized to meet the needs of older patients, and in collaboration between neurologic and geriatric teams to facilitate the transition of care. The management in the post-stroke geriatric unit is characterized by an overall assessment of the old patient, including detection and management of post-stroke complications [17, 27–30] and geriatric syndromes [31, 32]. Collaborative care involving geriatric teams and other specialized teams if a promising way to optimize care of very older patients. A large body of literature have reported experiments of collaborative care with geriatricians and cardiologists [33], oncologists [34] or surgeons [35, 36]. Observational studies carried out on these pathways are associated with better prognoses when older patients are hospitalized in collaborative pathways [35–37]. This suggests the importance of collaborative management of patients aged over 70 with chronic illnesses. One of the ways in which this can be achieved is through the creation of pathways like the SPOLD.

This study has some limitations. The level of evidence obtained by this observational study is much lower than that obtained in randomized controlled trials, even if we adjusted the outcome on the main prognosis factors for mortality after stroke. It is likely that the study lacked sufficient power to show a significant difference without adjustment. The cohorts are quite old. They were observed before COVID and fully functional as they tend to be after COVID. The principles and implementation of post-stroke rehabilitation have evolved but primarily through technological advances that have been sparsely implemented in most services in our sector [26]. In this study, the ongoing comprehensive geriatric care represents the true added value of the studied system. The CRW group was not homogeneous and patients’ management probably differed according the wards. We have noted that patients involved in this the SPOLD were older, and more complex and disabled, probably because the referral of these patients in the CRW is more difficult. Further observational studies of this original pathway are needed. Following the positive results of the first two observational studies on this pathway, the setting up of a randomized controlled trial should be discussed.

## Conclusion

Clinicians should consider starting collaborative care between geriatricians and neurologists to manage their patients over 70 years old to pathways such as SPOLD. Interestingly, collaborative care experiences between geriatricians and other specialties are on the rise and seem to benefit to the patient and to each medical member of those pathways. The studies on the SPOLD suggest that this pathway may be associated with a shorter length of stay, better survival and functional recovery. Geriatricians and neurologists wishing to improve the outcome of their post-stroke patients over 70 can more easily discuss the opening of a collaborative pathway such as SPOLD.

## Supplementary Information

Below is the link to the electronic supplementary material.

Supplementary file1 (PDF 299 KB)

Supplementary file2 (PDF 65 KB)

## Data Availability

The datasets generated and analyzed during the current study are available from the corresponding author on reasonable request. Due to the sensitive nature of medical data and in accordance with applicable regulations in France (such as GDPR), access may be granted following review by the ethics committee and/or the signing of a data use agreement.
