# Supplementary material for: Better survival of older patients with stroke managed in a collaborative stroke pathway
Source: Eur Geriatr Med. 2025 Jun 27;16(4):1551–8. doi: 10.1007/s41999-025-01225-9 (PMC12378616; doi:10.1007/s41999-025-01225-9)
Supplement: Supplementary file 1 — Supplementary file1 (PDF 299 KB) [file 41999_2025_1225_MOESM1_ESM.pdf]

Supplementary materal 1 : National Institutes of Health Stroke Scale scale (NIHSS scale)

NIH STROKE SCALE SHEET (Circle the Patient's Score)

Item	Score
**LOC**	0 = Alert, keenly responsive<br>1 = Not alert, but arousable by minor stimulation to obey, answer, or respond<br>2 = Not alert, requires repeated stimulation to attend, pain to move<br>3 = Responds with reflex motor, posturing, unresponsive, not movement, etc.
**Month Age**	0 = Answers both questions <u>correctly</u><br>1 = Answers one question <u>correctly</u>, intubated but follows command<br>2 = Answers neither question <u>correctly</u>, aphasic, stuporous, coma
**Open/Close Eyes Open/Close Good Hand**	0 = Performs both tasks <u>correctly</u> without coaching<br>1 = Performs one task <u>correctly</u>, even if weak<br>2 = Performs neither task <u>correctly</u>
**Best Gaze Horizontal (voluntary or Doll's)**	0 = Normal<br>1 = Partial gaze palsy, can be overcome by finger tracking/head turning<br>2 = Forced deviation or total gaze paresis, not overcome by the oculocephalic maneuver
**Visual Fields (upper, lower quadrants)**	0 = Normal, no visual loss, or regards/looks at moving fingers<br>1 = Partial (upper/lower quadrantopia) hemianopia, visual extinction<br>2 = Complete hemianopia<br>3 = Bilateral hemianopia, blindness
**Facial Palsy (show teeth, raise eye-brows, close eyes,use noxious stimuli)**	0 = Normal, symmetrical movement<br>1 = Minor asymmetry, ↓ nasolabial fold<br>2 = Partial paralysis lower face<br>3 = Complete paralysis 1 or both sides, upper/lower face (absence of facial movement in the upper and lower face)
**Motor Arm**	0 = **No drift;** limb holds 90 (or 45) degrees for full 10 seconds.<br>1 = **Drift;** limb holds 90 (or 45) degrees, but drifts down before full 10 seconds; does not hit bed or other support.<br>2 = **Some effort against gravity;** limb cannot get to or maintain (if cued) 90 (or 45) degrees, drifts down to bed, but has some effort against gravity.<br>3 = **No effort against gravity;** limb falls.<br>4 = **No movement.**<br>UN = **Amputation** or joint fusion, explain:__________<br>**5a. Left Arm** 0 1 2 3 4 UN<br>**5b. Right Arm** 0 1 2 3 4 UN
**Motor Leg**	0 = **No drift;** leg holds 30-degree position for full 5 seconds.<br>1 = **Drift;** leg falls by the end of the 5-second period but does not hit bed.<br>2 = **Some effort against gravity;** leg falls to bed by 5 seconds, but has some effort against gravity.<br>3 = **No effort against gravity;** leg falls to bed immediately.<br>4 = **No movement.**<br>UN = **Amputation** or joint fusion, explain:__________<br>**6a. Left Leg** 0 1 2 3 4 UN<br>**6b. Right Leg** 0 1 2 3 4 UN
**Limb Ataxia Out of Propor-tion to Weakness (test both arms FTN legs HTS)**	0 = Absent, global aphasia, complete hemiplegia<br>1 = Present in 1 limb<br>2 = Present in 2 limbs<br>UN = Post-angio maintain straight leg, amputation, joint fusion, explain:__________
**Sensory on Face/Arms/Legs (use pin or noxious)**	**Don't test on hands/feet**<br>0 = Normal, no sensory loss<br>1 = Mild to moderate sensory loss, can tell touch<br>2 = Severe to total sensory loss, not aware of touch, coma, or quadriplegic
**<u>Best Language</u> (describe picture, name objects, read sentences)**	0 = Normal, no aphasia<br>1 = Mild to moderate aphasia, can be understood<br>2 = Severe aphasia, much is not understood, excess listener work<br>3 = Mute, global aphasia, no usable speech or auditory comprehension, follows no 1 step commands, coma<br>**<u>Alternate method</u> if unable to read: repeat words/ sentences, object recognition after feeling object in hand**
**Dysarthria (clarity of articulation, read or repeat words)**	0 = Normal<br>1 = Mild to moderate dysarthria, some slurring, speech understood<br>2 = Severe, so slurred to be unintelligible<br>UN = Intubated, or other physical barrier explain:_______<br>**If patient is aphasic, judge only the spontaneous words**
**Extinction or Inattention, Neglect (test face and arms with bilateral simultaneous stimulation)**	0 = No abnormality<br>1 = Personal inattention or extinction to bilateral stimuli in any one of the senses (vision, tactile, auditory, spatial or personal)<br>2 = Profound inattention or extinction to more than one sense, may not recognize own hands or orients to only one side of space

TOTAL NIH STROKE SCALE SCORE ____________________
(Don't count any UN's in score)

DATE ________________ **TIME** ________________

EXAMINER __________________________
MD or RN (NIH Stroke Scale trained)

Please check appropriate box to indicate time this NIHSS completed:

❑ **Admit (or at time of Code 3 Stroke initiated)**
❑ **24 hours** ❑ **Discharge**

PLACE IN H&P/CONSULT SECTION OF CHART.

LABEL

SACRED HEART MEDICAL CENTER
Spokane, Washington
NIH STROKE SCALE

Approved by Neuroscience Service Line 9/17/04

9/04
#Z04570
