# Supplementary material for: Better survival of older patients with stroke managed in a collaborative stroke pathway
Source: Eur Geriatr Med. 2025 Jun 27;16(4):1551–8. doi: 10.1007/s41999-025-01225-9 (PMC12378616; doi:10.1007/s41999-025-01225-9)
Supplement: Supplementary file 2 — Supplementary file2 (PDF 65 KB) [file 41999_2025_1225_MOESM2_ESM.pdf]

Supplementary materal 2 : Modifed Rankin scale

**Modified Rankin Scale**
0 *point:* No symptoms
1 *point:* No significant disability, able to carry out all usual activities, despite some symptoms
2 *points:* Slight disability, able to look after own affairs without assistance, but unable to carry out all previous activities
3 *points:* Moderate disability, requires some help, but able to walk unassisted.
4 *points:* Moderately severe disability, unable to attend to own bodily needs without assistance, and unable to walk unassisted.
5 *points:* Severe disability requires constant nursing care and attention, bedridden, incontinent.
6 *points:* Dead.
